# A decade of progress in cancer research

**DOI:** 10.1186/1471-2407-11-498

**Published:** 2011-11-30

**Authors:** Christna Chap, Jigisha Patel

**Affiliations:** 1 BMC Cancer, BioMed Central, Gray's Inn Road, London, WC1 8HB, UK

## 

*BMC Cancer *[1] was launched ten years ago at a time when open access publishing was in its infancy. Although initially looked upon with skepticism, the open access publishing model, which provides an unrestricted and free access to scientific and scholarly work, has become increasingly accepted as a viable model, with more than 7, 000 open access journals now operating worldwide [2]. In a decade, this model has moved from the fringe to the core of the publishing industry.

*BMC Cancer *was one of the first journals to be open access and exclusively published online. The journal published 20 articles during its first year and has steadily grown since then with over 490 articles already published this year. *BMC Cancer *now boasts an impact factor of 3.15, which places it in the first quartile of the "oncology" category. To celebrate this first very successful decade for the journal, we present a special series of commissioned articles that not only highlight the most important advances in cancer research over the last ten years, but also discuss the new developments we might expect to see in the near future.

One major challenge in the treatment of cancer is to diagnose the disease at an early stage in order to improve prognosis. While several screening programmes exist for common cancers such as colorectal and breast cancer, they only detect cancers of one particular type [3,4]. In his commentary, **Ian Cree **[5] discusses how technical improvements in tests to detect circulating tumor markers, and indeed the potential for some molecules to act as general tumor markers, raise the possibility of blood test-based general screening for multiple cancers - in effect, screening for who to screen. Recently, an increasing number of studies have suggested that micro-RNAs (miRs) represent a promising new class of biomarkers for human malignancies [6-8]. In their review, **Fei-Fei Liu and colleagues **[9] specifically focus on the potential role of micro-RNAs as diagnostic and prognostic biomarkers in human epithelial tumors.

The last decade has also seen huge developments in sequencing technologies and several projects such as the Cancer Genome Atlas [10] and the International Cancer Genome Consortium [11] have aimed to provide a more complete picture of the mutational profile of cancer via large-scale analysis of cancer genomes. **Hans Kristian Moen Vollan and Carlos Caldas **[12] discuss how results of next generation sequencing hold the potential to refine molecular classification of breast cancer by integrating information from genomic signatures to existing knowledge derived from histopathological sub-classification. These advances could thus improve targeted cancer therapy and prognosis.

It is becoming increasingly clear that a multi-drug approach to targeted cancer therapy is needed due to increasing drug resistance to individual therapeutic agents [13,14]. In particular, **Joanna Pancewicz and Christophe Nicot **[15] discuss how recent advances on the role of Notch signaling pathway in the pathogenesis of human leukemia suggest that multi-drug chemotherapy targeting Notch signaling might be a promising therapeutic strategy in various hematological disorders.

In his commentary, **Dirk Vordermark **[16] discusses how the individualization of radiotherapy concepts and the combination of radiotherapy with molecular approaches are central to major developments in the field of radiation oncology, and he outlines the particular role of *BMC Cancer *as a platform for disseminating this research. Individualized and targeted therapy approaches hold great promise in oncology but resistance to therapy remains to be overcome. It is well-established that low tumor oxygenation can cause poor response to radiation therapy [17,18]. **Kevin Bennewith and Shoukat Dedhar **discuss in their review [19] how the clinical potential of hypoxic tumor cells extends beyond the treatment of primary tumors, and they elaborate on the promising role of hypoxic tumor cells as targets for the treatment and prevention of metastatic cancer.

This special issue sets the stage for 'hot topics' we might expect to see in the coming decade, and *BMC Cancer *is poised to be at the forefront of disseminating this research in a rapid and freely-accessible manner.

We would like to thank all our external academic editors and Editorial Board Members for their continuing support, and our Section Editors in particular for their contribution to this special anniversary issue.

We hope you enjoy reading it.

## Pre-publication history

The pre-publication history for this paper can be accessed here:

http://www.biomedcentral.com/1471-2407/11/498/prepub
